# Symptomatic Left-Sided Bochdalek Hernia in the Adult: A Case Report and Literature Review

**DOI:** 10.7759/cureus.93059

**Published:** 2025-09-23

**Authors:** Danny Tran, Hasin Sharma

**Affiliations:** 1 Internal Medicine, HCA Healthcare/University of South Florida (USF) Morsani College of Medicine Graduate Medical Education (GME) HCA Florida Trinity Hospital, Trinity, USA; 2 Osteopathic Medicine, Nova Southeastern University Dr. Kiran C. Patel College of Osteopathic Medicine, Clearwater, USA

**Keywords:** adult congenital diaphragmatic hernia, bochdalek hernia, conservative medical management, general surgery, hernia repair

## Abstract

A Bochdalek hernia, a type of diaphragmatic hernia, is a congenital hernia that typically presents on the left posterolateral diaphragm. It typically presents in early childhood as respiratory or gastrointestinal symptoms and diagnosis is confirmed by chest X-ray in pediatric patients. In many cases, treatment is surgical with mesh-based hernia repair. In children, surgical treatment is performed to prevent complications of pulmonary hypoplasia; whereas, in adults, surgical correction of the hernia is done for symptomatic management. We present a case of an 81-year-old female who came in with complaints of shortness of breath, nausea and vomiting, who was diagnosed with Bochdalek hernia on abdominal CT. Although a candidate for surgery, the patient decided to pursue conservative management. Our case emphasizes the importance of clinical suspicion and prompt imaging diagnosis in cases of older patients presenting with respiratory or gastrointestinal symptoms; this case also demonstrates management of Bochdalek hernia without surgical intervention.

## Introduction

Bochdalek hernias (BH), also known as pleuroperitoneal hernia, are a type of hernia that occurs in the posterolateral diaphragm. It is the most commonly occurring type of congenital diaphragmatic hernia; it comprises about 70-85% of all diagnosed congenital diaphragmatic hernias [1,2]. It has an incidence of about one in 2,000-3,000 live births [1]. Regarding the embryology of Bochdalek hernias, this is said to occur during the fifth week of gestation where the pleuroperitoneal membranes of the diaphragm form laterally, with the right side forming before the left side, to the diaphragm’s septum transversum (ultimately called the central tendon) [1]. When there is a defect in the formation of the pleuroperitoneal membranes of the diaphragm, this creates a thinned segment of the diaphragm or opening that causes a defect, and ultimately can allow abdominal contents to herniate into the thoracic cavity. This can lead to compression of the lungs which can lead to respiratory complications such as lung hypoplasia and pulmonary hypertension [2]. This can result in respiratory distress, as well as bowel strangulation and bowel ischemia [2]. 

Patients with Bochdalek hernias are generally pediatric patients who present with respiratory distress (nasal flaring, grunting), chest pain, and occasionally gastrointestinal symptoms such as vomiting, postprandial fullness and abdominal pain [3]. In adults, presentation is either asymptomatic or includes nonspecific respiratory and/or gastrointestinal symptoms [2,3]. Physical exam findings may include decreased or absent breath sounds in the affected side, bowel sounds heard in the lower lung lobes of the affected side and a scaphoid abdomen secondary to the herniation of gastrointestinal contents [2,3]. The majority of cases are diagnosed with abdominal ultrasounds in the neonatal period and are promptly surgically intervened upon to prevent complications. Asymptomatic cases can go undetected into adulthood until incidentally found or symptoms develop. 

There are several case reports that diagnose a symptomatic Bochdalek hernia in adulthood between ages 18 and 50 with almost all of them pursuing surgical repair [4-12]. In 1959, Kirkland details the rarity of adult cases which at the time totaled 34 [13]. However, with the advent and routine use of computed tomography (CT) scanners, the incidence of asymptomatic adult hernias has increased with two studies identifying almost 700 hernias upon retrospective review of CT scans within a one-year period [14,15]. In these studies, the patients had a mean age of about 60 and were all asymptomatic. All patients in the study by Kinoshita et al. did not undergo operative repair and were followed for 12 to 27 months. The study found that no significant sequential changes were observed during the follow-up period [15].

The current literature highlights a trend of operative repair in younger symptomatic patients and conservative management in older asymptomatic patients; however, we would like to present and discuss a case of an 81-year-old female who presented with a symptomatic left-sided Bochdalek hernia and wanted to pursue conservative management. 

## Case presentation

We present a case of an 81-year-old female with a medical history of hypertension, hyperlipidemia, and stage 4 chronic kidney disease, who presented to the emergency room with three days of nonspecific respiratory and gastrointestinal symptoms, including shortness of breath, nausea, vomiting, diarrhea, and lightheadedness.

**Figure 1:**
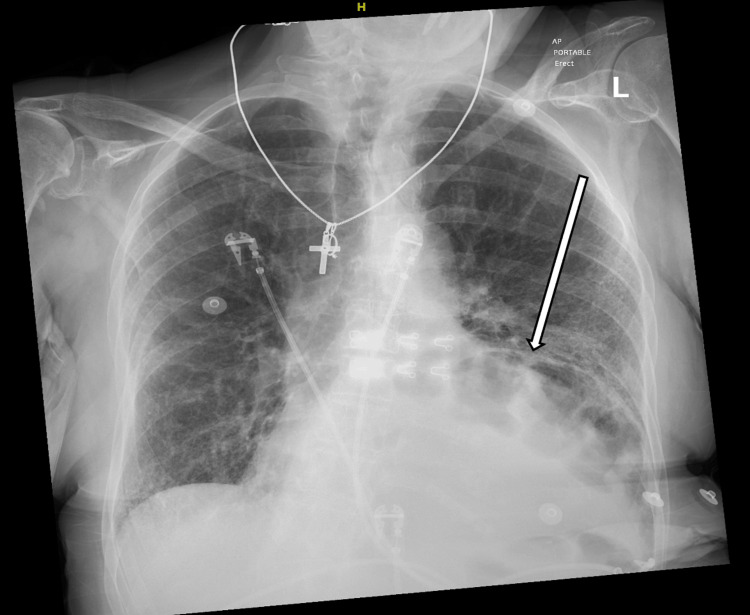
Posterior-Anterior Chest X-ray demonstrating bulging of the diaphragm on the left side and loops of bowel at the base of the left lung.

**Figure 2:**
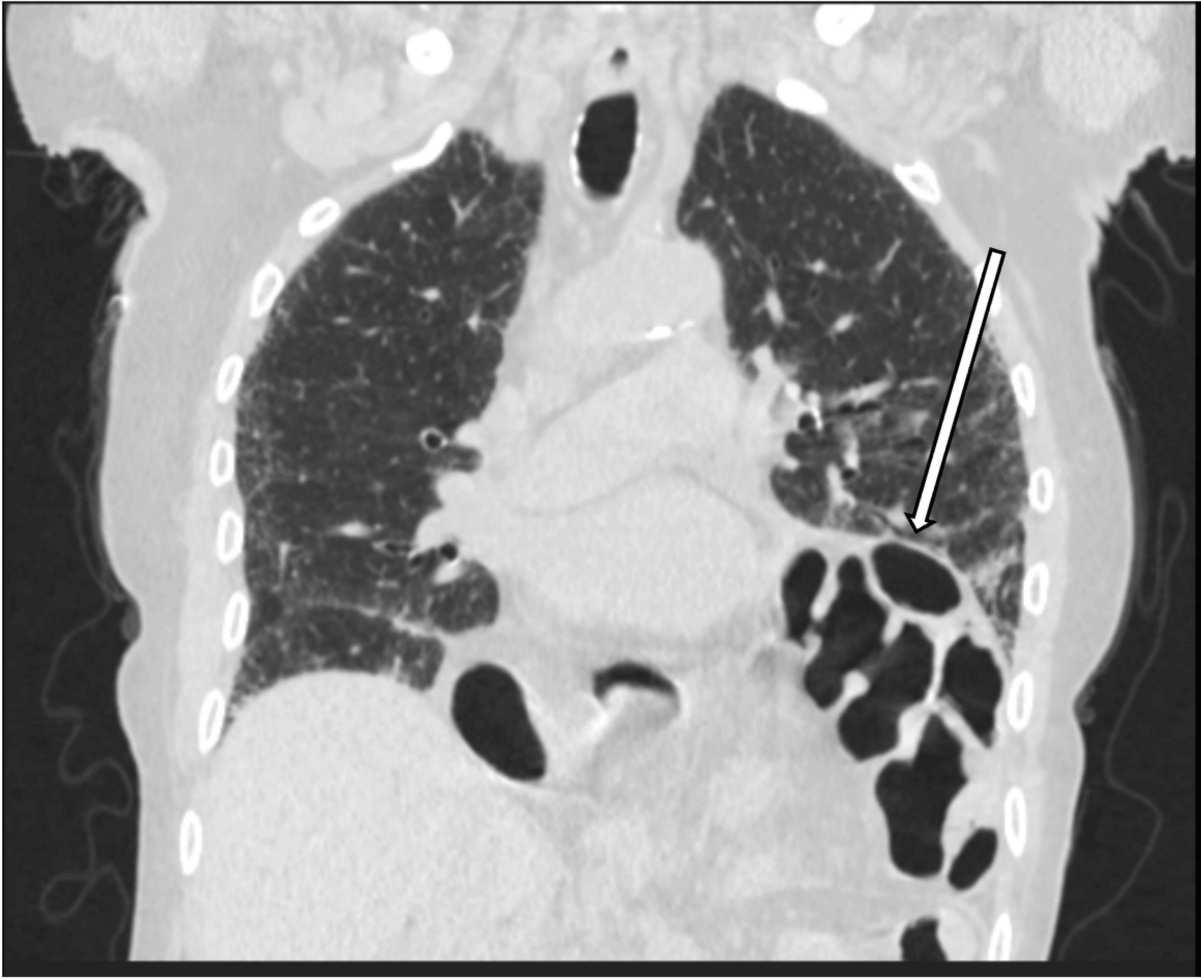
CT Chest Axial view in the lung window showcasing herniation of bowel contents into the left hemithorax.

**Figure 3:**
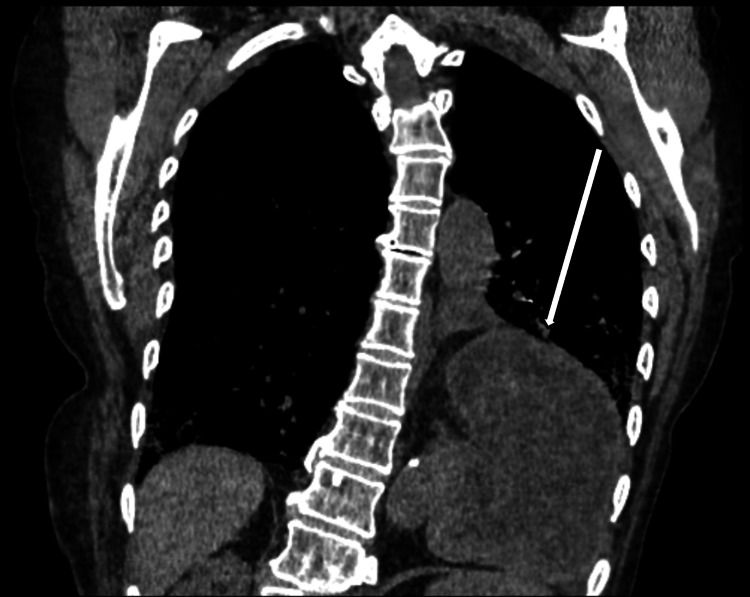
CT Chest Axial view showcasing herniation of the stomach into the left hemithorax.

**Figure 4:**
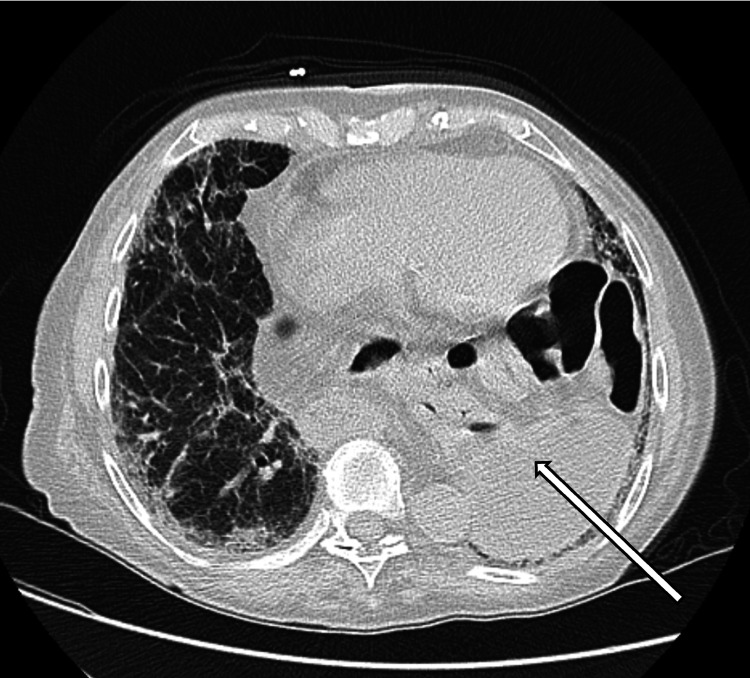
CT Chest Coronal view in the lung window showcasing the herniation of the stomach and bowel into the left hemithorax.

Evaluation in the ER revealed electrolyte abnormalities consistent with gastrointestinal losses (K 2.1, Cl 93, Na 128), lactic acidosis (lactic acid 3.4), and an elevated creatinine (Cr 3). Initial chest imaging, including a plain radiograph followed by CT of the chest, demonstrated a left-sided Bochdalek hernia with herniation of the stomach and small bowel loops, without evidence of bowel obstruction (Figures 1-4). The patient had no prior imaging available for us to review; she was unaware of the Bochdalek hernia until these results were discussed with her during hospitalization.

The patient was admitted, received intravenous fluids, and her nausea was treated with antiemetics, which corrected her electrolytes and improved renal function within the first 24 hours of hospitalization. The surgical team was consulted for the BH. They performed an esophagoduodenoscopy (EGD) to confirm viability of the stomach and the presence of the Bochdalek hernia. General surgery recommended mesh repair of the hernia given that the patient was symptomatic (vomiting and septic). They also recommended repair to avoid risk of future aspiration events. Cardiology was consulted for surgical clearance because she had new onset atrial fibrillation. They deemed her to be an intermediate risk for surgery but they cleared her if the surgery team determined that the benefits of surgery outweighed the risks.

However, upon sharing with the patient and family the team’s findings and recommendations, the patient expressed not wanting to undergo surgery. An extensive discussion was held with the patient and her family regarding her surgical indications and the potential sequelae of pursuing medical management including return of her presenting symptoms, abdominal organ incarceration and strangulation, risk of aspiration from the hernia, worsening respiratory status, and the possibility of severe morbidity and even death. Having acknowledged these risks, the patient continued to opt for conservative measures instead of treatment. The patient was ultimately discharged home with instructions to follow up with the surgeon outpatient and to return earlier if symptoms recur. 

Before she could follow up with the surgeon as an outpatient, the patient was readmitted weeks later for acute hypoxic respiratory failure secondary to an acute aspiration event from emesis. The aspiration event was determined to be related to the patient’s hernia so the surgeon recommended surgical repair of the hernia during her inpatient stay. The patient declined, and decided to have hospice services given her poor prognosis secondary to her chronic history, age and clinical status from the hernia. Ultimately, her clinical condition declined and she passed in hospice from hypoxia. 

## Discussion

This is a case of a symptomatic BH identified in adulthood. Typical management of BH involves surgical repair to minimize complications such as worsening respiratory symptoms and abdominal organ strangulation. This patient presented with respiratory complaints of shortness of breath and gastrointestinal symptoms of nausea, vomiting and diarrhea: the most common presentation associated with a Bochdalek hernia [16]. 

Furthermore, the abdominal CT revealed a Bochdalek hernia on the left side of the diaphragm - the left side tends to be the more common side due to the left canal of the diaphragm closing later than the right canal during gestation at around eight weeks [2]. In a setting where abdominal CT may not be available, one can utilize either an abdominal ultrasound or abdominal radiograph to diagnose BH. An ultrasound would reveal discontinuity and herniation of the viscera [2]. Meanwhile, an abdominal radiograph can indicate gas-filled loops of bowel, although sensitivity for detection is low [2]. Overall, diagnosis is mainly achieved by a combination of imaging, where most findings are incidental in nature, and of high clinical suspicion. Although Bochdalek hernias are quite rare in adult presentation, they tend to be misdiagnosed frequently. This can lead to a delayed diagnosis and consequently cause significant complications such as mesenteric ischemia if not promptly identified [17].

Regarding treatment, the first-line option is surgery. The purpose of surgery would be to prevent future complications from the BH, such as perforation of the bowels, bowel ischemia or hernia incarceration or obstruction [16]. If a case is emergent, an open laparotomy is preferred [18]. If a case is not emergent, or open repair is not feasible, it is possible to have an outpatient procedure with a laparoscopic approach; although, it is ideal that the surgery center should have extensive experience with such hernia repairs [18]. Thoracostomy is another procedure that could be done; however, it is primarily used in right-sided BH due to the hernia being masked by the liver. In terms of closures, most Bochdalek hernias can be closed with mesh repair (about 40%) or primary repair (about 50%) with more complications resulting from mesh repair [19]. Given that the hernia impacts the diaphragm, pulmonary complications such as pulmonary hypoplasia or obstructive lung disease should be considered. 

Of note, surgery is typically the first-line option in pediatric patients rather than elderly patients. Surgery was indicated for this patient because it was determined that she was symptomatic secondary to the hernia, which is an indication to surgery [20]. So for adult cases, the patient’s candidacy for surgery should be deemed on a case-by-case basis [20].

It is important to consider that depending on the patient’s comorbidities and cardiac risk under anesthesia, similar to our case, patients may not always be ideal surgical candidates. There are recorded cases where patients have been conservatively managed with symptomatic treatment and were deemed stable enough to be discharged without any symptoms at follow-up, but there are no established guidelines regarding a conservative approach to treatment, aside from follow-up imaging [16]. 

It is imperative that we account for such cases in the future by increasing initiatives on researching options for conservative treatment. Although the patient in this case was able to recover clinically and be safely discharged with extensive education about her condition, there may be other options that are safer and more effective that would warrant further research. 

## Conclusions

Depending on the presentation of Bochdalek hernias in the elderly population, they may range from an asymptomatic presentation to a life-threatening presentation with respiratory complications like our case presentation. The definitive treatment is a surgical hernia repair, once confirmation of the hernia is obtained through abdominal CT. If patients have a history of gastric symptoms, possible EGD may also be ideal to perform to confirm patency of the hernia and gastric surface. There may be cases where patients are not appropriate candidates for surgery, and may need to pursue conservative treatment. In cases where patients are elderly and/or possibly have other comorbidities, there may be a wish for comfort measures instead. Given this, surgical versus conservative options of treatment in adult and elderly patients should be considered on a case-by-case basis, with the patient's autonomy of highest regard. 
